# Research on scalable graphene faces a reproducibility gap

**DOI:** 10.1038/s41467-023-36891-5

**Published:** 2023-02-28

**Authors:** Peter Bøggild

**Affiliations:** Department of Physics, Technical University of Denmark, 2800 Kgs Lyngby, Denmark

**Keywords:** Peer review, Synthesis of graphene, Two-dimensional materials

## Abstract

More than a decade after the first demonstration of large-scale graphene synthesis by chemical vapor deposition, the commercialization of graphene products is limited not only by price, but also by consistency, reproducibility, and predictability. Here, the author discusses the reproducibility issues in the field and proposes possible solutions to improve the reliability of published results.

## Teaser

Science progress relies heavily on reproducibility and intersubjectivity. In science, intersubjectivity is when knowledge is communicated clearly between individuals and independently verified. It is the shared agreement and understanding of methods, results, and interpretations of scientific research that enables knowledge to be reviewed, revised, and established. Here, I focus on the reproducibility issues pertaining to the relatively young area of large-scale graphene synthesis and applications, proposing possible solutions to improve the reliability of published results and accelerate their commercialisation.

## Slow adoption of graphene technology

Since the first demonstrations of large-scale graphene synthesis^1^ by chemical vapor deposition (CVD) in 2009, the field has progressed through stages of wildly optimistic predictions, fierce competition, bold business ventures, and, to some degree, also disappointments. Despite of a large number of scientific papers on large-scale graphene and manufacturing-oriented technology, practical applications are still rare. The commercialization of CVD graphene products is limited not only by price, but also by consistency, reproducibility, and predictability. Also for graphene-based bulk materials (powders and solutions) the issue of variations in quality leading to mistrust and slow commercial adoption was discussed previously by Kauling et al.^2^ and Bøggild^3^. As an example, the 2D Experimental Pilot Line (2D-EPL), a 20-million Euro ecosystem of technologies and facilities in Europe, aims at establishing reproducible pilot line manufacturing and integration of 2D materials with silicon wafers. Professor Max Lemme (2D EPL steering group) explained that for this to succeed, standardized process modules and characterization, as well as statistically relevant amounts of data are crucial: “While original and exciting ideas are extremely valuable for exploring new concepts, reproducibility, standardization, and benchmarking are essential when translating science into production. This mindset should be reflected in scientific literature”.

## Thousands of graphene transfer methods

It *sounds* simple enough. Let me focus on one notorious aspect of large-area graphene: the transfer process. Graphene is grown on a catalytic substrate (often copper) by CVD, transferred to a target substrate, where further device fabrication and integration can proceed. In terms of fulfilling expectations for applications in electronics, optics, and barriers, the challenge is to carry out the transfer process rapidly, at a low cost, while maintaining the outstanding physical properties of a just 0.3 nm thin sheet of carbon atoms. The low dimensionality of the material leaves it highly susceptible to contamination, wrinkles, and interface roughness, all of which compromise the quality and consistency needed for disrupting anything.

Unsurprisingly, the transfer process turns out to be critically important, and unfortunately also delicate and difficult; after all, a graphene sheet is 40000 times thinner than plastic wrap. After the publication of nearly 3000 scientific articles on 2D material transfer, many of which claim to have solved the problem - partly or fully - the community is still intensively working on the matter. Why?

## “We followed all procedures, but it doesn’t work”

In our laboratory, we have faithfully contributed to the myriad of transfer methods, as well as attempted to reproduce transfer methods published in literature. However, we find that the latter is often surprisingly difficult, even when following the reported methodology to the letter. We often circumvent the problem by altering the method so much that we end up with a “new”, incrementally different method that we can then decide to publish in yet another article. Perhaps of more use for the community would be to report on our attempts to reproduce the original method. Such an article, however, is significantly harder to publish. Consequently, we get infinite variations instead of instant verification. A quick Google image search on “Graphene transfer process” effectively illustrates the issue of such incremental method mutations.

In 2016, we published an article^4^ in Nature Communications on the dry assembly of van der Waals materials, basically a method paper with little novel physics. We set out to make the description of the method as transparent, complete, and thorough as possible. We carefully reported on all samples and measurements, from the best to the worst, to give an honest picture of the performance and yield. One reviewer asked us to repeat the study with a new set of samples, which we did. We included all details that might help readers reproduce our results. To our surprise, we still received numerous requests for help from researchers having trouble reproducing the results. The leading postdocs, Bjarke Sørensen Jessen and Lene Gammelgaard, operated an ad-hoc helpdesk service for several years after publication of the article. Looking back, we could have achieved our original goals of transparency and reproducibility by asking independent groups to replicate our method before publishing the work.

## The power of independent verification

In general, the research papers we encounter in the field focus more on describing which procedures they followed, and less on guiding readers toward achieving similar results. This is a subtle but significant difference.

In a recent review that I carried out for a manuscript^5^ later published in Nature Communications, I raised concerns regarding the reproducibility of work from an excellent, highly reputed research group at Beijing University. The quality of the data was impressive, but I was uncertain whether colleagues could achieve similar results based on the article. I was worried that the outstanding results presented in the manuscript could be the result of a very well-oiled machine – the mastery of the complex fabrication and characterisation steps - rather than the superiority of the specific method in question. As it happened, the authors of the manuscript responded to the criticism by documenting thoroughly that an independent research group had indeed reproduced the work. Apart from immediately putting my concerns to rest, it struck me how seldom I see anything like this. Imagine how much more powerful “methods”-oriented publications would be if they routinely communicated acceptance windows, robustness, operation ranges, consistency, yield, and repeatability in a clear, standardized, and systematic manner. If more articles tried to establish and document intersubjectivity, such as for instance the article by Cheng et al.^6^ on how to report and benchmark emerging field effect-transistors, individual fields would evolve more steadily and rapidly.

## Updating the science game rule book

These considerations are part of a wider debate that followed an article^7^ entitled “1500 scientists lift the lid on reproducibility” published in Nature in 2016. The article sparked a staggering number of 1700 citations and an altimetric score of 5000. Additionally, 80% of the respondents felt that funders and publishers should do more to improve reproducibility.

In line with the respondents to the Nature survey^7^, I feel that the rule book of the funding and publishing game should be updated to encourage or even enforce a higher degree of reproducibility, and that this could have a lasting impact on the field and the technology everybody is waiting for. COARA, launched by the European Union in 2022, seeks to reform research assessment in evaluations of individual researchers as well as organizations. This initiative has hundreds of funding institutions already signed on. One passage in the agreement reads: “Openness of research, and results that are verifiable and reproducible where applicable, strongly contribute to quality. Openness corresponds to early knowledge and data sharing, as well as an open collaboration including societal engagement where appropriate.” The agreement aims to alter the rules of the massive game that modern research in my view has become. Another example is the Validation’s reproducibility initiative, which seeks to reward and identify high-quality reproducible research via independent validation of key experimental results. It was founded by, among others, Mendeley and PLOS. The Brazilian Reproducibility Initiative goes the extra mile by repeating between 60 and 100 published experiments, each in three different laboratories throughout the country^8,9^. A number of initiatives like this exist across a wide variety of research fields; perhaps the most famous is the 8-year Reproducibility Project: Cancer Biology (RPCB), which reported that half of top cancer studies failed to be replicated^10^.

## Hardwiring reproducibility into publications

Clearly, publishers and editors of high-impact journals are central stakeholders in the symbiotic relationship between scientists and distributors of science. Imagine a novel form of scientific publication, where reproduction studies are actively encouraged or required in analogy to how many publishers require data availability statements. If an article had a formal reproducibility statement with extensive details on yield, robustness, guidelines, and results of independent verification, this would be without question scrutinized by any researcher attempting to build on the research. Original journal publications may be linked to other studies that reproduce them and vice versa. Active discussion threads where researchers could share reports on their efforts to verify the the published results, or assist colleagues with guidance, would be highly desirable. For this to work, the efforts of reproducing research need to be praised, credited, and recognized. Imagine if along with the main paper, a handful of brief, concise reports from independent groups on reproducibility and yield were available. Over the past 15 years of 2D material research in my team, such a practice could have saved us years of reinvention, reverse engineering, and dead ends.

Despite various reproducibility efforts, I believe that the lack of support for reproducibility and inter-subjectivity is a structural problem of the scientific community that holds several negative consequences: a focus on news value over quality, a slowing of scientific progress, and finally a decrease in adoption of innovative technologies. The industry cannot stay on standby while researchers continue to invent incrementally different solutions.

## Better reproducibility can fuel disruptive research

Paradoxically, the hunt for novelty over reproducibility could be the enemy of disruptive research as well as technological progress. A study published in Nature in 2023 stated that the proportionality of disruptive ideas per scientist has been steadily declining^11^; that fewer and fewer papers reveal radical advances in knowledge that challenge existing paradigms. In this study, “disruption” was measured by the CD index^12^: the more groundbreaking an article is, the less the subsequent citing articles would cite previous work.

These ideas have been around for a while. A decade ago, Simonton argued^13^ in Nature that “scientific genius is extinct”, as research progresses in larger and larger teams with fewer original ideas. I believe that the core values of intersubjectivity not only support but precede disruptive research. A truly sharp idea may well be overlooked in the haystack of incremental, “micro-original” articles. An idea only becomes disruptive, if it is noted in the sea of noise, and reproduced.

By now, after multiple reports and extensive media attention, most researchers and publishers must be aware of these issues. The question is whether it is possible to update the reward system of the “science game”, most researchers play. If so, how, and when? And by whom? The disruptive shift that occurs when novel information challenges established knowledge cannot take place without intersubjectivity. If we don’t understand, acknowledge and respect how knowledge is formed, we will not be able to bow to it. The common ground of the scientific community is based on shared language, standards, norms, methods - and reproducibility.

## References

[1] Li XS (2009). Large-area synthesis of high-quality and uniform graphene films on copper foils. Science.

[2] Kauling AP (2018). The worldwide graphene flake production. Adv. Mater..

[3] Boggild P (2018). The war on fake graphene. Nature.

[4] Pizzocchero F (2016). The hot pick-up technique for batch assembly of van der Waals heterostructures. Nat. Commun..

[5] Gao X (2022). Integrated wafer-scale ultra-flat graphene by gradient surface energy modulation. Nat. Commun..

[6] Cheng ZH (2022). How to report and benchmark emerging field-effect transistors. Nat. Electron..

[7] Baker M (2016). 1,500 scientists lift the lid on reproducibility. Nature.

[8] Amaral OB, Neves K, Wasilewska-Sampaio AP, Carneiro CF (2019). Science Forum: The Brazilian Reproducibility Initiative. Elife.

[9] Neves K (2020). Two years into the Brazilian Reproducibility Initiative: reflections on conducting a large-scale replication of Brazilian biomedical science. Mem. Inst. Oswaldo Cruz.

[10] Errington TM, Denis A, Perfito N, Iorns E, Nosek BA (2021). Reproducibility in cancer biology: Challenges for assessing replicability in preclinical cancer biology. Elife.

[11] Park M, Leahey E, Russel JF (2023). Papers and patents are becoming less disruptive over time. Nature.

[12] Funk RJ, Owen-Smith J (2017). A dynamic network measure of technological change. Manage. Sci..

[13] Simonton DK (2013). After Einstein: Scientific genius is extinct. Nature.

